# Exploring Novel Technologies in Lung Cancer Diagnosis: Do We Have Room for Improvement?

**DOI:** 10.7759/cureus.6828

**Published:** 2020-01-31

**Authors:** Munish Sharma, Salim Surani

**Affiliations:** 1 Internal Medicine, Corpus Christi Medical Center, Corpus Christi, USA; 2 Internal Medicine, Texas A&M Health Science Center, Bryan, USA

**Keywords:** lung cancer, pulmonary nodules, low dose computed tomography, bronchoscopy, gene expression classifier

## Abstract

Lung cancer remains the leading cause of cancer-related death worldwide. Preventive strategies, mainly smoking cessation have a big impact on the reduction of lung cancer-related mortality. Screening with low dose computed tomography (LDCT) has proven to be beneficial in reducing the mortality related to lung cancer mainly based on early detection of cancer and timely initiation of treatment. Despite its beneficial effects, guideline-directed LDCT screening could lead to high false positive results, subjecting patients to harmful radiation, increase cost of healthcare and induce anxiety amongst the patients. Thus, it is imperative to look beyond the prevailing modalities of lung cancer screening and diagnosis to achieve better yield and mitigate the existent drawbacks.

## Introduction and background

With the surge in awareness regarding detrimental effects of cigarette smoking, developed nations, in particular, have witnessed some downward trend in lung cancer but it still remains a major cause of mortality and morbidity globally with 2.1 million cases and 1.8 million deaths reported in the year 2018 [1]. In the United States, lung cancer occurs in around 230,000 patients and causes more than 140,000 deaths every year [2]. There were 310,000 new cases and 265,000 deaths due to lung cancer in the European Union in 2012 [3]. It was estimated to cost $21.3 billion in lost earnings in the U.S in 2018, which accounted for 22.5 % of overall lost earnings from all the cancers [4]. Thus, it is obvious that preventive strategies along with effective screening methods are imperative to cause any sort of dent to this massive financial burden, inflicted by lung cancer. In this context, we hereby attempt to evaluate the current understanding regarding lung cancer screening and diagnosis and assess newer technologies that could be adjunctive tools to conventional diagnostic modalities in lung cancer.

## Review

The current state of lung cancer diagnosis

The diagnosis of lung cancer is not always made in the earlier stages, which can affect the possible treatment and cure. There are many inherent characteristics of lung cancer that justify the embracement of the screening strategy. Significant mortality and morbidity associated with the advanced stage of lung cancer, easily identifiable major risk factors to target high-risk population and prolonged pre-clinical phase of certain types of lung cancer (non-small cell lung cancers as compared to small cell) necessitate timely initiation of screening strategy [5]. Also, it is evident that treatment strategies are more effective in the early stage of the disease to achieve a better cure rate [6].

Efforts to devise the best screening strategy for lung cancer has been an area of research for past decades. Seven large scale clinical trials involving radiograph alone or radiograph combined with sputum cytology were studied, but it failed to show any significant mortality benefits in patients with a high risk of lung cancer [7,8]. Subsequently, the National Lung Screening Trial (NLST) attempted to shed some answers. They conducted a three-year study on 53,454 high-risk patients in the U.S [9,10]. It compared low dose computed tomography (LDCT) with a chest radiograph showing a clear mortality benefit. Based on this trial, it is recommended to use LDCT for lung cancer screening in patients aged 55 to 74 years with at least 30 pack-years of smoking history including active smokers and those who quit smoking within the past 15 years [9-11]. Nodules found with the aid of LDCT are classified based on Lung CT Screening Reporting and Data System (Lung-RADS) [12]. This reporting system provides a crude estimate of malignancy and helps to guide a clinician in managing patients with lung nodules. Lung-RADS recommends the continuation of annual LDCT even if no nodules were detected in the initial scan as a part of lung cancer screening guidelines in the high-risk individuals. Solid nodules <6 mm are followed yearly, pure subsolid nodules <20 mm are followed yearly while those ³ 20 mm are followed every six months [12]. 

Incidentally detected pulmonary nodules can account for a significant number of lung cancer diagnoses. For such incidental nodules, further surveillance and diagnosis are based on Fleischner guideline 2017 [13]. It divides pulmonary nodules into solid and subsolid lesions. Part solid and pure ground-glass lesions are further subtypes of subsolid nodules. Single and multiple solid nodules < 6 mm and low-risk category do not need a routine to follow up while those considered to be in the high-risk category only need follow up computed tomography (CT) chest at 12 months. Solid single nodule of 6-8 mm (low and high risk) requires CT at 6-12 months, followed by 18-24 months. Multiple 6-8 mm nodules should be followed up with CT chest at 3-6 months than at 18-24 months. Solid nodules >8 mm (both single and multiple) require CT at 3 months, positron emission tomography (PET)/CT or biopsy [13]. Sub solid nodules <6 mm do not require routine follow up unless there are multiple nodules in which case CT at 3-6 months, two years and four years is recommended. Sub solid ground glass nodules > 6 mm should undergo CT at 6-12 months then at three and five years. Part solid nodules > 6 mm require to follow up CT at 3-6 months and then yearly for five years if persistent (Table 1) [13].

**Table 1:** Fleischner society guideline 2017 for pulmonary nodules [13] CT: Computed tomography, PET: Positron emission tomography.

Type of pulmonary nodule	Size of nodule	Follow up		
Solid	< 6 mm	Single nodule	Low risk /High risk	No routine follow up/CT at 12 months-optional
		Multiple nodules	Low risk/High risk	No routine follow up/CT at 12 months-optional
	6-8 mm	Single nodule	Low risk/High risk	CT at 6-12 months, then at 18-24 months/CT at 6-12 months, then at 18-24 months
		Multiple nodules	Low risk/High risk	CT at 3-6 months, then at 18-24 months/CT at 3-6 months, then at 18-24 months
	>8 mm	Single nodule	Low risk/High risk	CT at 3 months, PET/CT or biopsy
		Multiple nodules	Low risk/High risk	CT at 3-6 months, then at 18-24 months/CT at 3-6 months, then at 18-24 months
Ground glass	<6 mm	No follow up recommended		
	> 6 mm	CT at 6-12 months->if persistent, CT at 3 and 5 years		
Part-solid	< 6 mm	No follow up recommended		
	> 6 mm	CT at 3-6 months->if persistent, yearly CT for 5 years		
Multiple	< 6 mm	CT at 3-6 months, if stable size CT at 2 and 4 years		
	> 6 mm	CT at 3-6 months and management thereafter based on characteristics of most suspicious nodule		

Challenges of current guidelines in lung cancer screening 

There are several pitfalls in existing guidelines for lung cancer screening and further surveillance. Patients with lung nodules may undergo an invasive biopsy that might ultimately yield a benign result. Such invasive modalities can have their own associated mortality and morbidity [14,15]. As evident in the NLST, 95% of the pulmonary nodules were false-positive and 11% of positive results led to invasive testing [11]. Data from the Veterans Health Administration showed that 73 (3.5%) of 2106 patients who underwent lung cancer screening were subjected to further invasive testing and follow up imaging. Only 31 patients (1.5%) of all the patients screened finally proved to have lung cancer [16]. The risk of radiation exposure is another significant concern. Effective radiation dose with LDCT is 1.4 millisieverts (mSV), which is less than 7 to 8 mSV for standard CT chest [17]. Moreover, it has been estimated that cumulative radiation dose over a period of 10 years is 13 mSV in women and 9.3 mSV in men. Thus, one major cancer would be caused by radiation for every 108 patients with the diagnosis of lung cancer [17]. Due to the prolonged course for follow up of lung nodules that may often last for years, there might be short-term distress in patients [18]. Physicians involved in such rigorous process may have their own issues with uncertainty hovering around their patient’s final diagnosis. Prevailing strategy in screening also raises issues of “over-diagnosis” in patients who are already at increased risk for other causes of mortality and morbidity [19]. Cost-effectiveness is another major factor that is often drawn into the debate. An analysis performed before the conclusion of NLST, estimated the cost to be US $ 126,000 to $ 269,000 per quality of adjusted life year (QALY) for lung cancer screening to obtain a reduction in lung cancer mortality by up to 25% at 10 years [20]. Another study estimated the cost to be around $81,000 per QALY [21]. Thus, for the current screening strategy, which has around 95% false-positive results and relatively low number of deaths prevented (73/100,000 person-years), cost-effectiveness does seem to be a factor [22].

Exploring new avenues in lung cancer screening and diagnosis

Identification of tumor biomarkers can help in the early detection of small-sized lung cancers before they can be visualized on radiographs. If lead-time bias can be ruled out, such tumor biomarkers can potentially achieve superior outcomes [23]. Biomarkers can be obtained from epithelial cells of the respiratory tract, exhaled breath or blood sample [23]. Immunostaining or automated image cytometry of sputum, analysis of volatile organic compounds in exhaled breath and genomic analysis of samples obtained from bronchoscopy are some newer technologies that are currently under investigation [24-27]. Airway Epithelial Gene Expression in the Diagnosis of Lung Cancer (AEGIS) 1 and 2 were two multicenter prospective trials conducted in 28 centers that included a total of 639 patients (298 in AEGIS-1 and 341 in AEGIS-2). It studied if bronchial-airway gene-expression classifier could be used to improve bronchoscopy directed diagnostic yield for lung cancer [28]. Under the receiver-operating-characteristic curve (AUC) for the gene expression classifier was 0.78 (95% confidence interval of 0.73-0.83) in AEGIS-1 and 0.74 (95% confidence interval of 0.68-0.80) in AEGIS-2. Sensitivity was 88% in AEGIS-1 and 89% in AEGIS-2. Thus, based on the results of AEGIS-1 and 2, physicians can decide to pursue a more conservative approach if gene expression classifier is negative in patients with intermediate-risk and non-diagnostic bronchoscopy [28]. Though there are various predictive models for solitary pulmonary nodules to be malignant such as the Mayo Clinic model [29], no validated models were used for patients undergoing diagnostic bronchoscopy in this study [28]. Patients were selected for bronchoscopy examination based on a qualitative assessment of the physicians. This study had a follow up of 12 months duration as opposed to 24 months recommended in the standard guideline for solitary lung nodule but the conversion rate of lung nodules, which remain negative at 1 year follow up is only 1 per 1000 in the second year [28]. This study excluded lifetime non-smokers and those with a history of already established lung cancer. Hence, lung cancer in non-smokers and recurrence in those with a history of tumor resection is not addressed by AEGIS-1 and 2 [28]. In a separate study conducted to assess the cost-effectiveness of the use of gene expression classifiers and bronchoscopy versus bronchoscopy alone, the total costs and QALY gain were similar to the use of classifier and resulted into incremental cost-effectiveness ratio at 3% per year [29].

Molecular and protein-based tumor markers obtained from a simple blood test can also be utilized in the context of incidentally diagnosed pulmonary nodules that are likely to be benign [30]. This might help physicians to categorize patients in low to moderate risk groups for malignancy, instead of subjecting them to invasive testing. Silvestri and colleagues in PANOPTIC study [30] utilized the blood-based molecular test for incidental lung nodules ranging between 8 to 30 mm in size and a pre-test risk of malignancy less than or equal to 50% determined by solitary pulmonary nodule calculator [31]. This test utilized the mass spectrometry to identify two circulating proteins and coupling with clinical risk factors to satisfy the proprietary algorithm to obtain a final result with up to 97% sensitivity and 98% negative predictive value [30]. This test is covered by Medicare in the USA with no out of pocket expenses [32].

PET with fluorodeoxyglucose (FDG) after LDCT performed on an annual basis for evaluation of non-calcified lung nodules ³ 7 mm diameter were studied in two separate studies. Sensitivity was 69%, specificity was 91%, positive predictive value was 90% and the negative predictive value was 71% for PET-FDG [33]. Repeat CT at an interval of 3 months after negative PET-FDG was able to obtain 100% negative predictive value [33]. In a study performed on 172 patients with NSCLC who underwent CT Chest and PET/CT, the incremental cost-effectiveness ratio for every patient correctly staged was $3,508 in the case of PET/CT as compared to CT only [34]. There is some scope of incorporating PET-FDG in future radiological imaging screening algorithm for lung cancer if cost-effectiveness is not a limiting factor.

Assessment of tumor growth pattern was evaluated in The Continuous Observation of Smoking (COSMOS) study to determine if the volume doubling time (VDT) or growth rate of the lung tumors could be used to determine indolent cancers. Tumors with VDT of more than 400 days likely represented overdiagnosis and could have been managed with a conservative approach [35].

The innovations involving novel and cutting-edge technology and biomarkers will gain wider acceptance only when they are tested rigorously in well-designed clinical trials. Data regarding their effectiveness in early diagnosis of lung cancer, cost-effectiveness and an overall reduction in lung cancer mortality will generate additional enthusiasm in the clinicians for their utility. These techniques carry the potential to be game-changers in lung cancer screening and diagnosis. 

## Conclusions

Future endeavors in lung cancer screening and diagnosis must be geared towards finding a more personalized approach. Newer non-invasive adjunctive tests, based on a better understanding of genomic expressions in lung cancer can play a pivotal role in the future. These newer diagnostic modalities can potentially help evaluate the patients judiciously, on a case-by-case basis for appropriateness of further invasive testing. This can improve the yield of current diagnostic modalities, reduce the humongous burden on healthcare cost and will likely help alleviate uncertainty associated with prevailing diagnostic methods and their results. The anxiety that often hovers in patients' and physicians' minds during the tedious and protracted process of lung nodule and ground-glass opacities and subsequent diagnosis needs to be eliminated.
